# An Infant With COVID-19 Presenting With a Bulging Fontanel: A Case Report and Literature Review

**DOI:** 10.7759/cureus.63667

**Published:** 2024-07-02

**Authors:** Hiroyuki Kuroda, Yoshiki Kusama, Ayu Ogura, Takashi Matsunaga, Yukari Atsumi, Katsunori Kamimura

**Affiliations:** 1 Department of Pediatrics, Hyogo Prefectural Amagasaki General Medical Center, Amagasaki, JPN; 2 Department of Infectious Diseases, Osaka University Hospital, Suita, JPN

**Keywords:** antimicrobial stewardship, infant health, bulging fontanelle, pediatric meningitis, covid-19

## Abstract

A bulging fontanel is a sign of elevated intracranial pressure, which can be caused by diseases with intracranial fluid retention or swelling of the cerebral parenchyma. We experienced a case of an infant with a typical course of mild coronavirus disease 2019 (COVID-19) but with a bulging fontanel as a finding at presentation. The patient, a three-month-old boy with no underlying conditions, presented to the emergency clinic with fever, vomiting, and loss of appetite. Due to the absence of crying and the bulging fontanel, he was referred to our hospital with suspected bacterial meningitis. The diameter of the anterior fontanel was 2.5 cm, as measured by the Popich and Smith method. He showed no signs of consciousness impairment and appeared to be as active as usual. Computed tomography revealed a bulging fontanel. Cerebrospinal fluid examination showed no elevated cell counts, and cultures were negative. Accordingly, bacterial meningitis was ruled out. The fever resolved on the day after admission, and the patient was discharged on the third day after admission in good general condition. When an infant diagnosed with COVID-19 presents with a bulging fontanel, it is important to be aware of its low specificity and excessive antibiotic treatment should be reconsidered.

## Introduction

A bulging fontanel is a sign of elevated intracranial pressure, which is caused by diseases with intracranial fluid retention or swelling of the cerebral parenchyma [1]. There is no clear definition of a bulging fontanel, but it can be identified by palpating the bulge and touching the bone in the vestibular area. Meningitis is a well-known infectious disease that can cause bulging fontanel, but this symptom can also be caused by other infections, such as roseola infantum [2]. We experienced the case of an infant who presented with a typical course of mild COVID-19 but had a bulging fontanel at presentation. Although it is difficult to prove a bulging fontanel on physical examination due to the lack of an objective assessment and modification of the symptom by crying [3], in the present case, it was demonstrated on computed tomography (CT). Since there are few case reports of COVID-19 with bulging fontanel, we report this case along with a literature review of previous cases.

## Case presentation

Case

A three-month-old boy with no notable medical history presented to the emergency clinic with poor feeding, a single episode of vomiting, and a fever. He was referred to our hospital with suspected meningitis due to the absence of crying and a bulging fontanel. COVID-19 was diagnosed based on a positive qualitative SARS-CoV-2 antigen test at the clinic. He had been born via cesarean section at 37 weeks and four days gestation without apparent asphyxia, with a birth weight of 2,564 g. No maternal infections were noted prenatally, and the mother’s group B Streptococcus (GBS) screening test was negative. The mother had not consumed cured ham or cheese during pregnancy. The boy had received the Hib vaccine twice, the pneumococcal vaccine twice, the hepatitis B vaccine twice, and the tetanus, diphtheria, and pertussis vaccines twice. His mother had been diagnosed with COVID-19 17 days before his hospital visit.

History before admission

**Figure 1:**
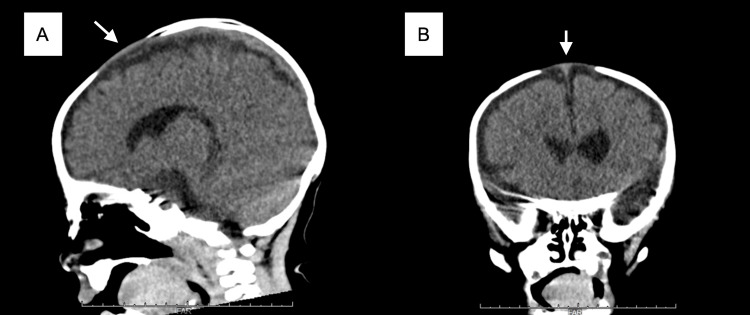
Computed tomography findings Sagittal (A) and coronal (B) reconstruction showed a bulging fontanel. The images show the projection of the large fontanel from the skull (arrows).

At the emergency department, the patient’s vital signs were as follows: body temperature, 39.0℃; heart rate, 178 beats/min; respiratory rate, 40 breaths/min; and oxygen saturation, 97% on room air. The capillary refilling time was less than two seconds. In the dorsal position, a physician was unable to follow the entire circumferential bone edge of the anterior fontanel that was suspected to be bulging. The diameter of the anterior fontanel was 2.5 cm, as measured by the Popich and Smith methods, which determine the size by averaging the width and length of the fontanel. He showed no signs of consciousness impairment and appeared to be as active as usual. The patient’s skin color and muscle tone were normal. Sucking and grasping reflexes in both the palms and soles were intact. There were no findings of sunset eye signs or other neurological abnormalities, such as paralysis. There were no abnormalities of breath or heart sounds. The patient’s respiratory pattern was stable, and there was no suggestion of circulatory failure. A projection of the large fontanel from the skull was discovered on computed tomography, which was conducted to rule out cerebral bleeding and mass lesions. There were no space-occupying or hemorrhagic lesions, hydrocephalus, or bone fractures (Figure 1).

Considering the possibility of meningitis, cefotaxime (100 mg/kg) was administered after taking a blood culture. Laboratory analysis revealed the following (Table 1): white blood cell count, 5.8 × 10^9^/L; C-reactive protein, 0.24 mg/dL; procalcitonin, 0.13 ng/mL; and serum glucose, 10^9^ mg/dL. There were no organ damage or electrolyte abnormalities, except mild elevation of aspartate aminotransferase (74 IU/L) and alanine aminotransferase (65 IU/L). This mild liver damage was considered attributable to COVID-19. Lumbar puncture revealed a white blood cell count of 1.0/mm^3^, a protein level of 13.7 g/dL, and a glucose level of 57 mg/dL. These laboratory data did not indicate meningitis.

**Table 1:** Results of laboratory tests AST: aspartate aminotransferase, ALT: alanine aminotransferase, CSF: cerebrospinal fluid, ND: not determined

Test	Unit	Case	Reference
Leukocyte count	10^9^/μL	5.8	3.3-8.6
Percentage of neutrophils	%	39.3	43-65
Percentage of lymphocyte	%	38.0	20-50
Red blood cell count	10^12^/μL	4.35	4.35-5.55
Hemoglobin	g/dL	11.4	13.7-16.8
Thrombocyte count	10^9^/μL	216	158-348
Total billirubin	mg/dL	0.6	0.4-1.5
AST	IU/L	74	13-30
ALT	IU/L	65	10-42
Urea nitrogen	mg/dL	6.6	8.0-20
Creatinine	mg/dL	0.26	0.65-1.07
Creatine kinase	IU/L	130	59-248
Glucose	mg/dL	109	73-109
C-reactive protein	mg/dL	0.24	0.00-0.14
Procalcitonin	ng/mL	0.13	< 0.5
Sodium	mmol/L	138	138-145
Potassium	mmol/L	4.7	3.6-4.8
Chloride	mmol/L	107	101-108
Total cell count (CSF)	/μL	1	0-5
Total protein (CSF)	mg/dL	13.7	8-43
Glucose (CSF)	mg/dL	57	ND

Differential diagnosis

Cerebral hemorrhage, brain tumor, brain abscess, hydrocephalus, meningitis, head trauma, and viral infection were included as differential diseases since a bulging fontanel occurs due to elevated intracranial pressure.

Clinical course

Due to the patient's young age of three months and the presence of fever and vomiting, it was deemed necessary to rule out bacterial meningitis. We decided to treat the child with a regimen for bacterial meningitis until blood and cerebrospinal fluid cultures were confirmed to be negative for bacteria. On admission, the patient was treated with cefotaxime and vancomycin. Forty-eight hours after admission, blood and cerebrospinal fluid cultures were negative, and antibiotic medication was discontinued. On the day after admission, the patient’s fever subsided, and his general status improved. He was discharged on the third day of hospitalization. Three days after discharge, the patient was reassessed in the outpatient department and was found to be well. Follow-up laboratory analysis and imaging were not performed to avoid unnecessary invasive procedures and radiation exposure.

## Discussion

We reported the case of a three-month-old infant with COVID-19 who presented with a bulging fontanel but no evidence of meningitis and who had a mild natural course. Although bulging fontanel is a well-known finding suspicious for meningitis, Shacham et al. reported in 2008 that only one case of bacterial meningitis occurred among 153 infants presenting with fever and bulging fontanel and they raised questions about the need for routine CSF testing in infants with these symptoms [3]. In a more recent study, Takagi et al. reviewed 304 febrile patients of 2-18 months of age with bulging fontanel and reported that 40% had upper respiratory tract infection, 25% had aseptic meningitis, 9.8% had some type of viral infection, and only 0.3% had bacterial meningitis [4]. Freedman et al. pointed out that vaccination may be a cause of bulging fontanel [5], and Amarilyo et al. reported that the sensitivity and specificity of bulging fontanel for bacterial meningitis were 50% and 60%, respectively, with a positive predictive value of only 38% during a period when pneumococcal and Hib vaccines were widely available [6]. Therefore, bulging fontanel should not be considered the sole determining factor in the diagnosis of meningitis, but should be combined with other physical and laboratory findings.

At the beginning of the COVID-19 pandemic, cases in infants were rare, but the emergence of the Omicron strain made it more prevalent in infants [7]. Nevertheless, to our knowledge, only three cases of COVID-19 patients with bulging fontanel have been reported in the literature [8-10] (Table 2).

**Table 2:** Reported cases of COVID-19 with a bulging fontanel

Reported year	Authors	Age	Sex	Lumbar tap	Brain imaging	Treatment	Hospital stay
2021	Schiff et al. [9]	4 month	Girl	Done	CT	Ceftriaxone	Not hospitalized
2022	Al Amri et al. [10]	6 month	Girl	Done	Ultrasonography	Ceftriaxone	Not hospitalized
2023	Sethuraman et al. [8]	9 month	Boy	Done	CT and MRI	Ceftriaxone, clarithromycin, and acyclovir	5 days
This case		3 month	Boy	Done	CT	Cefotaxime and vancomycin	3 days

All three cases were treated with antibacterial or anti-herpesvirus medication, although imaging and CSF examinations were performed with no evidence of meningitis. These patients fully recovered with a typical mild course, consistent with COVID-19. Evaluating and treating fever in an infant who appears well is a challenging issue. The American Academy of Pediatrics guidelines state that, in infants older than 29 days of age with an unknown source of infection, there is no need for CSF testing if there is no elevated inflammatory response on a blood test [11]. Despite this recommendation, if there are signs that suggest meningitis, such as a bulging fontanel, it would be difficult to choose not to perform a CSF test, as it is the only way to confirm the presence of meningitis [12]. However, in the absence of elevated cell counts on CSF testing, the appropriateness of empiric antimicrobial administration should be debated. Antimicrobial treatment during infancy leads to the acquisition of drug-resistant bacteria. Future allergic diseases such as bronchial asthma, atopic dermatitis, and obesity have also been noted [13,14]. If the likelihood of meningitis is clearly low based on a physical examination and laboratory findings, it may be possible to observe the patient without administering antimicrobial medication. However, no studies have examined the need for antibiotic medication in infants with a bulging fontanel who are otherwise well. In fact, antibiotics have been used in all previous cases of infants with COVID-19 with a bulging fontanel. Further research is necessary to discuss the appropriateness of observing infants without medication in such cases.

## Conclusions

While a bulging fontanel is a finding that requires attention and may suggest bacterial meningitis, it can also be observed in infants with common viral infections, including COVID-19. When an infant diagnosed with COVID-19 presents with a bulging fontanel, it is important to be aware of its low specificity, and excessive antibiotic treatment should be reconsidered.

## References

[1] Kiesler J, Ricer R (2003). The abnormal fontanel. Am Fam Physician.

[2] Cristoforo T, Le NK, Rye-Buckingham S, Hudson WB, Carroll LF (2020). The not-so-soft spot: pathophysiology of the bulging fontanelle in association with roseola. Pediatr Emerg Care.

[3] Shacham S, Kozer E, Bahat H, Mordish Y, Goldman M (2009). Bulging fontanelle in febrile infants: is lumbar puncture mandatory?. Arch Dis Child.

[4] Takagi D, Oren-Ziv A, Shles A, Schujovitzky D, Yechiam H, Rosenbloom E (2021). Bulging fontanelle in febrile infants as a predictor of bacterial meningitis. Eur J Pediatr.

[5] Freedman SB, Reed J, Burwen DR, Wise RP, Weiss A, Ball R (2005). Transient bulging fontanelle after vaccination: case report and review of the vaccine adverse event reporting system. J Pediatr.

[6] Amarilyo G, Alper A, Ben-Tov A, Grisaru-Soen G (2011). Diagnostic accuracy of clinical symptoms and signs in children with meningitis. Pediatr Emerg Care.

[7] Bahl A, Mielke N, Johnson S, Desai A, Qu L (2023). Severe COVID-19 outcomes in pediatrics: an observational cohort analysis comparing Alpha, Delta, and Omicron variants. Lancet Reg Health Am.

[8] Sethuraman C, Holland J, Priego G, Khan F, Johnson R, Keane M (2023). Bulging anterior fontanelle caused by severe acute respiratory syndrome coronavirus-2. Pediatr Infect Dis J.

[9] Schiff J, Brennan C (2021). Covid-19 presenting as a bulging fontanelle. Am J Emerg Med.

[10] Al Amri MD, Helal O, Suliman A (2022). Bulging fontanelle as a sign of COVID-19 infection in infant: a case report. Medicine.

[11] Pantell RH, Roberts KB, Adams WG (2021). Evaluation and management of well-appearing febrile infants 8 to 60 days old. Pediatrics.

[12] Beri S, Hussain N (2011). Bulging fontanelle in febrile infants: lumbar puncture is mandatory. Arch Dis Child.

[13] Stocker M, Klingenberg C, Navér L (2023). Less is more: antibiotics at the beginning of life. Nat Commun.

[14] Vangay P, Ward T, Gerber JS, Knights D (2015). Antibiotics, pediatric dysbiosis, and disease. Cell Host Microbe.

